# Consequences of a changing US strategy in the global HIV investment landscape

**DOI:** 10.1097/QAD.0000000000001669

**Published:** 2017-11-09

**Authors:** Jessica B. McGillen, Alana Sharp, Brian Honermann, Gregorio Millett, Chris Collins, Timothy B. Hallett

**Affiliations:** a Department of Infectious Disease Epidemiology, Imperial College London, London, UK; b amfAR, The Foundation for AIDS Research; c Friends of the Global Fight Against AIDS, TB and Malaria, Washington DC, USA.

**Keywords:** Africa, economics, global health investment, global health policy, mathematical models

## Abstract

**Objective:**

The global fight against HIV/AIDS in Africa has long been a focus of US foreign policy, but this could change if the federal budget for 2018 proposed by the US Office of Management and Budget is adopted. We aim to inform public and Congressional debate around this issue by evaluating the historical and potential future impact of US investment in the African HIV response.

**Design/methods:**

We use a previously published mathematical model of HIV transmission to characterize the possible impact of a series of financial scenarios for the historical and future AIDS response across Sub-Saharan Africa.

**Results:**

We find that US funding has saved nearly five million adults in Sub-Saharan Africa from AIDS-related deaths. In the coming 15 years, if current numbers on antiretroviral treatment are maintained without further expansion of programs (the proposed US strategy), nearly 26 million new HIV infections and 4.4 million AIDS deaths may occur. A 10% increase in US funding, together with ambitious domestic spending and focused attention on optimizing resources, can avert up to 22 million HIV infections and save 2.3 million lives in Sub-Saharan Africa compared with the proposed strategy.

**Conclusion:**

Our synthesis of available evidence shows that the United States has played, and could continue to play, a vital role in the global HIV response. Reduced investment could allow more than two million avoidable AIDS deaths by 2032, whereas continued leadership by the United States and other countries could bring UNAIDS targets for ending the epidemic into reach.

## Abstract 2

Supplemental Digital Content is available in the text

The worldwide AIDS response has achieved enormous progress over the past 15 years. Concerted efforts by international donors and affected countries to scale up treatment and prevention programming have led to a critical point: if the global financial response continues to grow, a 90% reduction in AIDS-related deaths relative to 2010 is achievable within the next 15 years [1]. But of the 37.6 million people worldwide who were still living with HIV in 2015, over half lacked access to lifesaving antiretroviral therapy [2]. If donor countries pull back from the global response now, this opportunity to reach a turning point will be lost, risking backsliding into a resurgent epidemic.

In the landscape of global health and development expenditures [3], the United States is the largest donor to the fight against HIV by virtue of the President's Emergency Plan for AIDS Relief (PEPFAR) and contributions to the Global Fund to Fight AIDS, Tuberculosis, and Malaria. Proposed in 2003 by President George W. Bush, PEPFAR fundamentally altered the course of the HIV epidemic and is currently supporting nearly 11.5 million people on antiretroviral treatment in developing countries. The United States is also the biggest contributor to the Global Fund, the world's largest public–private funder for HIV, tuberculosis, and malaria. PEPFAR and the Global Fund are complementary, interreliant, and vital leaders of the global AIDS response [4].

Recently, the US Office of Management and Budget proposed a federal budget blueprint for the fiscal year of 2018 that reduces funding to foreign aid and states an intention to provide ‘sufficient resources to maintain current commitments and all current patient levels on HIV/AIDS treatment under PEPFAR’ [5].

Several studies have described the impact of US investments in stabilizing or reducing HIV incidence, but many are retrospective, qualitative, small in scope, or focused on individual funding streams or interventions [6–9]. Most substantive are the ongoing Population-based HIV Impact Assessments Project, which in 2016 reported decreasing HIV incidence in three PEPFAR-recipient countries [10], and a *Lancet* commission that estimated the costs of several global health investment scenarios [11]. However, uncertainty regarding US global health investments warrants an examination of both the investment counterfactual and the projected long-term impact of changes to the US strategy – broad policy questions for which mathematical modeling is a powerful tool. We apply a mathematical model [12] (summarized in Appendix pg. 1–3) to build a comprehensive picture of the impact of US investment across Sub-Saharan Africa, which accounts for 70% of the global HIV burden and where many countries have insufficient domestic resources to meet long-term treatment needs [13]. We describe how the HIV epidemic might have progressed in Sub-Saharan Africa if the United States had failed to invest, how the epidemic is likely to evolve under several scenarios for future US funding decisions, and explore the relative impact of a shift away from key at-risk populations.

Since 2003, the Global Fund and PEPFAR have funded treatment delivery, supported testing and counseling services, played a role in lowering the cost of antiretroviral drugs, scaled up HIV prevention by spearheading voluntary medical male circumcision and other prevention programs, and encouraged implementing countries to increase their domestic HIV spending. We constructed a historical counterfactual using financial data to depict the loss of these contributions if PEPFAR and the Global Fund were never founded (Appendix pg. 3–6). It was assumed in this case that behavior change initiatives would have been limited, domestic investments lower, and antiretroviral therapy persistently expensive. Although it is possible that US investment has suppressed, rather than encouraged, increases in domestic contributions [14], this remains under debate [15,16] and governments might have faced high opportunity costs in attempting to increase domestic HIV spending by redirecting funds from other health initiatives. Under this worst-case counterfactual, the model shows that mortality would not have begun to decline in the mid-2000s but instead would have continued to increase. This would have directly resulted in 3.7 million more HIV infections by the end of 2016, and nearly five million more people dead of AIDS-related causes, relative to what has occurred (Fig. 1a–c).

**Fig. 1:**
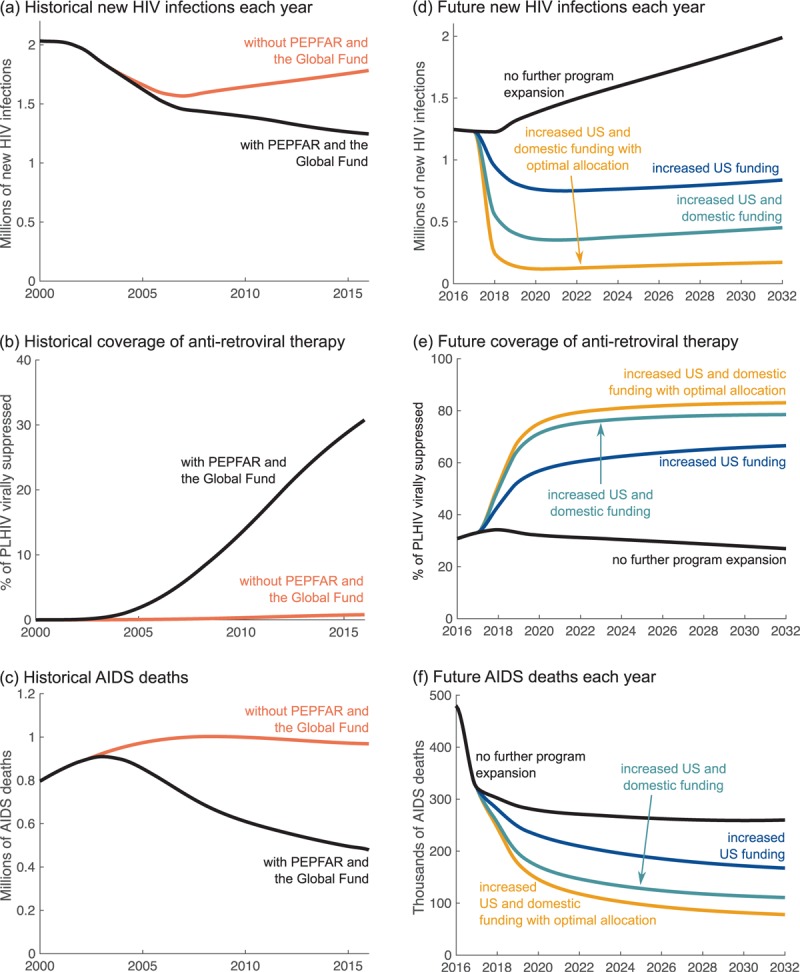
Modeled impact of US leadership in the AIDS response in Sub-Saharan Africa under historical (a–c) and future (d–f) scenarios.

PEPFAR, President's Emergency Plan for AIDS Relief; PLHIV, people living with HIV.

Sustained progress toward global targets set by the Joint United Nations Program on HIV/AIDS (UNAIDS) [1] will require the continued rapid expansion of antiretroviral therapy in concert with the prevention of new HIV infections [17]. Three factors will drive the impact that can be achieved by future treatment and prevention efforts: the amount of funding contributed by the United States and other donors, the degree to which governments of implementing countries increase their domestic financing, and policies determining how available resources are spent. We have developed a series of modeling scenarios to explore these drivers (Appendix pg. 7–12). The results are summarized in Table 1 and Fig. 1d–f.

**Table 1:** Summary of scenarios for future HIV funding decisions.

	No further program expansion^a^	Increased US funding^b^	Increased US and domestic funding^c^	Increased US and domestic funding with optimal allocation^d^
Total funding from all sources, 2017–2032	$81 billion	$116 billion	$146 billion	$146 billion
% of PLHIV^e^ virally suppressed on treatment by 2032	28%	66%	77%	83%
Total HIV infections, 2017–2032	25 700 000	13 200 000	7 360 000	3 520 000
Total AIDS deaths, 2017–2032	4 360 000	3 330 000	2 460 000	2 040 000
New HIV infections in 2032	1 990 000	838 000	453 000	173 000
AIDS deaths in 2032	260 000	167 000	111 000	78 000

^a^Maintenance of current numbers of people on treatment in all locations without further scaling up of coverage or introduction of new prevention interventions (Appendix pg. 8).

^b^A 10% increase in yearly US funding from the present level, with other international contributions remaining flat at present levels and conservative domestic projections in which domestic public HIV spending in the modeled countries increases in line with economic growth (Appendix pg. 8–11).

^c^A 10% increase in US funding, other international funding remaining flat, and ambitious domestic projections which see modeled countries boosting their HIV spending to match a benchmark based on the HIV share of the disease burden (Appendix pg. 11–12).

^d^The same overall budget as in the previous scenario (footnote 3), but with allocation to geographies, population groups, and interventions being responsive to local epidemiology (Appendix pg. 12).

^e^PLHIV, people (adults age 15 and above) living with HIV.

As one option, the present number of people on antiretroviral therapy could be maintained with no further expansion of treatment or prevention programs (Appendix pg. 8). This would cost an estimated $81 billion over 2017–2032 and the total percentage of adults living with HIV who are virally suppressed on treatment would decline from 33% in 2017 to 28% by 2032. Treatment has the important secondary benefit of preventing ongoing transmission, but this impact would be reduced with declining coverage, and the annual number of new HIV infections would increase by 43% relative to the number seen in 2010. This would allow a total of 4.4 million AIDS deaths over the 15-year period. Compared with the number of deaths in 2010, this would be a reduction of 57% by the end of the 15-year period – far short of the UNAIDS target of a 90% reduction over the same length of time.

As a second scenario, the United States could increase investment by 10% in PEPFAR and the Global Fund, while maintaining current spending patterns. In this scenario, we assume that other international funds remain flat, funding is distributed to countries according to present patterns, governments of implementing countries grow their own HIV spending only in line with economic trends, and prevention interventions are rolled out nationally (Appendix pg. 8–10). In this case, we estimate that the total HIV funding envelope will be $116 billion over the next 15 years. This will enable provision of treatment to 66% of adults living with HIV and avert 12.5 million HIV infections and more than one million AIDS deaths relative to no expansion of programs. Compared with 2010 numbers, these are reductions of 40 and 73% in new HIV infections and AIDS deaths, respectively.

Alternatively, the 10% increase in United States spending may be accompanied by governments of implementing countries increasing their own domestic HIV spending to meet an ambitious target developed by Resch *et al.*[18] and based on the HIV share of the disease burden (Appendix pg. 10–11). In this case, the total HIV budget will be $146 billion, enabling 77% of adults living with HIV to be virally suppressed on treatment and averting 18 million HIV infections and 1.9 million AIDS deaths relative to no further program expansion. These are reductions of 67% in HIV infections and 82% in AIDS deaths compared with 2010 numbers. Thus, against a backdrop of increased US funding, governments of affected countries can avert 44% more infections and 26% more deaths by moving from status-quo to ambitious HIV spending. However, even this level of response will not reach UNAIDS targets without improvements in how funds are allocated to population groups and prevention interventions.

Our final scenario explores an idealized HIV response whereby the United States both increases its present investment level by 10% and leads a concerted effort to optimize the channeling of prevention resources. In this case, funds are allocated to prevention portfolios that are optimized to subnational epidemiology (maximizing infections averted for the lowest cost) and specifically target key at-risk populations (Appendix pg. 11–12). This is in line with PEPFAR's ‘right things, right places, right time’ policy of making high-impact programs responsive to the people at greatest risk and in high-burden places [19]. For a funding envelope of $146 billion, we find that this approach will enable 83% of adults living with HIV to be virally suppressed on treatment and avert 22 million HIV infections and 2.3 million AIDS deaths over the 15-year period, relative to no further program expansion. These are reductions on 2010 levels of 88% for HIV infections and 87% for AIDS deaths. This strategy comes closest by the end of the 15-year period to achieving the UNAIDS targets of 95–95–95 for treatment (95% of people living with HIV knowing their status, 95% of those diagnosed receiving treatment, and 95% of those receiving treatment having viral suppression) and 90% reductions in new infections and deaths relative to 2010 numbers [1]. Using investments optimally (Appendix pg. 11–12) can thus avert 52% more infections and 17% more AIDS deaths than the same amount of funding used less efficiently.

These findings are supported by a large body of cost-effectiveness studies that indicate good returns on HIV investment in many settings (for example, [20–22]). Nevertheless, the numbers presented here should be interpreted with caution, as considerable uncertainty underlies our modeling assumptions (Appendix pg. 12), the data on which the model is based, estimates of intervention costs, how other countries might react to a changing US strategy, and unforeseen events that might affect future decisions of all countries. Furthermore, this model does not address the potential externalities of United States and other donor aid which are likely to produce impact beyond that achieved by HIV treatment and prevention interventions alone. These include poverty reduction, improvements in national stability and security, and health systems strengthening [23]. We have, therefore, likely underestimated the impact of US investments in HIV. We also note that the impact described herein may be achieved by multiple donor countries and implementing governments, rather than by a unilateral increase in US investments.

Our validated model has enabled us to measure both the historical impact of US investment and its plausible future impact under several scenarios – and we have found that if the United States and other donors choose to amplify their HIV response in concert with strong domestic support in developing countries, the world can approach UNAIDS targets for ending the epidemic. This analysis contributes one piece of a larger decision process which enables policy makers to synthesize a wide range of evidence in a formal way, and will, we hope, help to inform public debate and the choice facing those who will decide the US foreign aid HIV budget and strategy going forward.

## Acknowledgements

A.S., B.H., G.M., and C.C. conceived the study; all authors designed the study; J.B.M. did the analysis and wrote the paper; all authors edited the paper and approved the final version. J.B.M. and T.B.H. thank the Bill & Melinda Gates Foundation for funding this work through a grant to the HIV Modelling Consortium at Imperial College London.

### Conflicts of interest

There are no conflicts of interest.

## Supplementary Material

Supplemental Digital Content
