# Supplementary material for: Consequences of a changing US strategy in the global HIV investment landscape
Source: AIDS. 2017 Nov 9;31(18):F19–23. doi: 10.1097/QAD.0000000000001669 (PMC5690304; doi:10.1097/QAD.0000000000001669)
Supplement: Supplemental Digital Content [file aids-31-0f19-s001.doc]

Consequences of a changing U.S. strategy in the global HIV

investment landscape

*JB McGillen, A Sharp, B Honermann, G Millett, C Collins, TB Hallett*

**Supplementary Appendix**

Contents

**1 Overview of our approach 1**

**2 Model of HIV transmission in sub-Saharan Africa 1**

Figure S1: Modeled HIV prevalence at 10-year intervals 2

Figure S2: Model agreement with historical UNAIDS data 3

**3 Estimates of current HIV financing 3**

Table S1: Current HIV funding from all sources 4

**4 Analysis of the historical impact of U.S. investment 5**

4.1 The historical HIV response 5

4.2 Counterfactual with no U.S. investment 5

Figure S3: Estimated domestic HIV spending in 2002 6

**5 Future prevention interventions and their unit costs 7**

Table S2: Available prevention interventions 7

**6 Analysis of future U.S. policy options 8**

6.1 No further expansion of programs 8

6.2 Increased U.S. funding (with status quo otherwise) 8

Figure S4: Domestic spending breakdown 9

6.3 Increased U.S. and domestic funding 10

Table S3: HIV/AIDS burden by country 11

6.4 Increased U.S. and domestic funding with optimal allocation 11

**7 Key assumptions and limitations of this study 12**

**8 References (24–39) 13**

1 Overview of our approach

We employ a mathematical model of the HIV epidemic in sub-Saharan Africa to test a variety of strategies for responding to the epidemic. The use of mathematical modeling enables us to depict the historical epidemic trend, explore a counterfactual trend, and also assess the impact a given strategy might have on the future trajectory of the epidemic.

This Appendix first describes our model of HIV in sub-Saharan Africa in Section 2. In Section 3, we present estimates of current HIV/AIDS financing that will underpin the analyses developed here. Section 4 outlines the modeled historical trajectory and a counterfactual of no U.S. investment. Section 5 summarizes unit costs we use for future prevention interventions. Options for U.S. policies going forward are developed as modeling scenarios in Section 6. Finally, Section 7 discusses key assumptions and limitations behind the study.

2 Model of HIV transmission in sub-Saharan Africa

Full details of the model structure employed here, its parameters, and their calibration to data can be found in the recent paper by McGillen et al. [12]. The core of this framework is a dynamic compartmental model that describes sexual HIV transmission among adults (ages 15+), deaths, treatment, and prevention. We model each top-level administrative subnational region in 18 countries (Benin, Botswana, Burkina Faso, Cameroon, Congo, Democratic Republic of the Congo, Ethiopia, Kenya, Mali, Mozambique, Nigeria, Rwanda, Sierra Leone, South Africa, Swaziland, Tanzania, Zambia, and Zimbabwe), in total capturing 80% of the burden by people living with HIV in sub-Saharan Africa according to UNAIDS estimates [24]. Aggregated model outputs are multiplied by a factor of 1/0.8 to extrapolate from the modeled countries to all of sub-Saharan Africa.

In each subnational region, the population of adults is grouped by risk level. Key high-risk populations are female sex workers (FSW) and men who have sex with men (MSM). The general population includes low-risk women and men, who tend to form stable long-term partnerships; moderate-risk women and men, who form casual partnerships; and high-risk male clients of sex workers. Heterosexual HIV transmission links men and women, homosexual HIV transmission links MSM, and risk structure further differentiates how the population groups interact and how HIV is transmitted among and between them. After HIV infection, the model tracks disease progression by CD4 cell count status.

Parameters governing sexual behaviors and population demographics have been calibrated for each subnational region to produce epidemic dynamics consistent with local data on prevalence, circumcision, demographic characteristics of population groups, and historical treatment and prevention initiatives. Parameters governing biological aspects of HIV (such as disease progression rates) are held fixed across subnational regions. The calibration is detailed fully in the Supplemental Appendix of the paper by McGillen et al. [12].

The validated model describes a likely spatiotemporal trajectory of the HIV epidemic across sub-Saharan Africa that is consistent with available historical data (Fig. S1). There is broad agreement between aggregate model numbers and available UNAIDS data (Fig. S2), with differences arising from the underlying model structure and assumptions. Our model is at the subnational, rather than national, level and contains a mechanistic description of HIV transmission among and within risk groups, rather than assuming uniformity of risk. Additionally, our model considers sexual transmission between adults only and contains two age groups (age 15–49 and 50+) but otherwise is not age-structured.


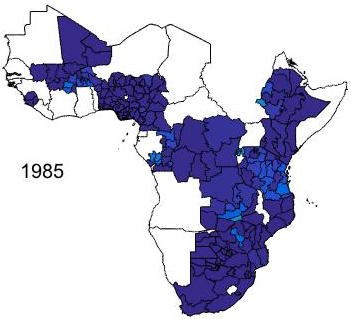

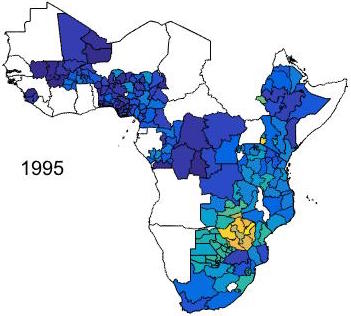
**
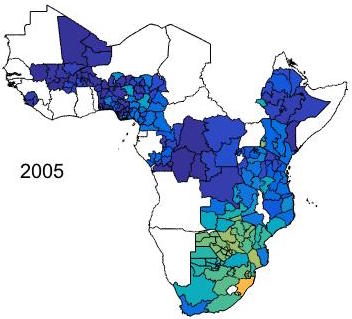
**


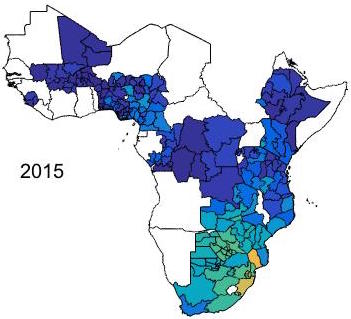

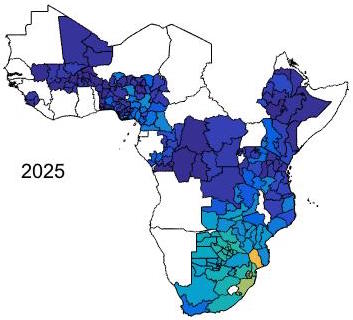

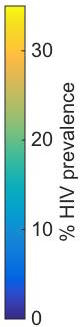


**Figure S1: Modeled HIV prevalence at 10-year intervals**, consistent with available historical epidemic data, as described in the recent paper by McGillen et al. [12].

B

A

C

D

**Figure S2: Model agreement with historical UNAIDS data.** Aggregate model results (black) against historical UNAIDS data (blue) for the (A) number of new HIV infections each year, (B) number of people living with HIV, (C) number of people on antiretroviral therapy (ART), and (D) number of AIDS-related deaths, all age 15+. Here, the future model trajectory shown assumes no intervention beyond continued scale-up of ART.

3 Estimates of current HIV financing

As will be described in Sections 4 and 6, this study is based around estimates of the current amount of HIV funding from all sources. Due to limitations of data availability, we use the most recent possible data points as proxies for current spending levels. Funding sources are PEPFAR, the Global Fund (with donations coming from both the U.S. and other countries), domestic public HIV spending, and other international funding. Private funding is assumed to be negligible in comparison to these larger funding channels. Table S1 summarizes all 'current' financial data by source and modeled country.

Total available PEPFAR funding amounts by country or regional program were extracted from the Office of the U.S. Global AIDS Coordinator’s quarterly reports to Congress [25]. As only cumulative amounts are described in the reports, quarterly funding amounts were estimated by subtracting amounts from previous quarters. Several countries not in the original set of PEPFAR focus countries received funding that only reported post-2008 as one lump sum. For these countries, annual funding from previous years was estimated as a proportion of the first reported year’s funding. As reports to Congress are not publicly available for 2015, the 2015 funding estimates were taken from the planned amounts in the Country Operational Plans for COP15 [26].

Global Fund disbursement amounts in the most recent available year (2006 for Botswana and 2014 for all other countries) were estimated using Aidspan’s database [27]. Amounts were summed over HIV and HIV/TB components. United States proportional support to the Global Fund replenishment is reported by the Global Fund for replenishment periods 2001-2007, 2008-2010, 2011-2013, and 2014-2016 [28].

Estimates of domestic public spending on HIV/AIDS (in $USD) were obtained from UNAIDS [24]. Data availability varies by country, with the most recent available data years ranging from 2005 to 2014.

A current estimate of other international resources was calculated using total HIV expenditures from National AIDS Spending Assessment (NASA) country reports via UNAIDS [24] in 2014, with the 2014 values for all other sources (PEPFAR, Global Fund, and domestic disbursements) subtracted. For some countries the resulting values are zero, likely due to data limitations such as underestimation of total HIV spending in some NASA reports.

**Table S1: Current HIV funding from all sources**. All funding amounts are in $USD. Countries have zeros in the PEPFAR disbursement column if they are not PEPFAR focus countries. PEPFAR estimates are from 2015; Global Fund disbursements are from 2014 for all countries except for Botswana, which is from 2006; other international estimates are from 2014; and domestic public HIV expenditures are from the listed data year. DRC=Democratic Republic of the Congo.

| Country | PEPFAR disbursements | Global Fund disbursements | | Other international  spending | Domestic public  HIV spending | |
| --- | --- | --- | --- | --- | --- | --- |
|  |  | From U.S. | Other donors |  | Amount | Year |
| Benin | 0 | 8,874,300 | 23,326,000 | 0 | 3,936,233 | 2014 |
| Botswana | 38,963,672 | 447,150 | 1,146,400 | 49,590,000 | 299,022,611 | 2011 |
| Burkina Faso | 0 | 8,791,600 | 23,108,000 | 15,112,000 | 7,780,061 | 2013 |
| Cameroon | 30,919,040 | 3,582,800 | 9,417,200 | 3,175,800 | 14,805,716 | 2013 |
| Congo | 0 | 2,172,900 | 5,711,300 | 2,703,700 | 8,104,228 | 2010 |
| DRC | 51,524,208 | 7,165,600 | 18,834,000 | 68,351,000 | 2,710,269 | 2012 |
| Ethiopia | 175,000,000 | 26,651,000 | 70,049,000 | 109,590,000 | 54,448,264 | 2011 |
| Kenya | 489,236,800 | 44,096,000 | 115,900,000 | 0 | 153,454,537 | 2013 |
| Mali | 0 | 6,366,400 | 16,734,000 | 978,360 | 6,136,480 | 2012 |
| Mozambique | 330,523,488 | 24,143,000 | 63,457,000 | 0 | 12,217,622 | 2014 |
| Nigeria | 405,198,848 | 57,876,000 | 152,120,000 | 0 | 122,964,880 | 2012 |
| Rwanda | 71,285,704 | 25,962,000 | 68,238,000 | 80,076,000 | 19,946,470 | 2012 |
| Sierra Leone | 0 | 5,015,900 | 13,184,000 | 7,088,900 | 168,584 | 2011 |
| South Africa | 300,095,712 | 37,482,000 | 98,518,000 | 0 | 1,492,672,908 | 2014 |
| Swaziland | 47,112,696 | 1,716,700 | 4,512,400 | 5,545,900 | 33,155,127 | 2013 |
| Tanzania | 412,049,760 | 65,042,000 | 170,960,000 | 0 | 108,120,344 | 2005 |
| Zambia | 313,297,344 | 35,001,000 | 91,999,000 | 0 | 15,829,478 | 2012 |
| Zimbabwe | 95,000,000 | 24,032,000 | 63,168,000 | 59,412,000 | 34,347,820 | 2013 |

4 Analysis of the historical impact of U.S. investment

We used the financial data in Section 3 to characterize a counterfactual investment scenario in which the U.S. did not exhibit leadership in the early 2000s, leaving the AIDS response to proceed without PEPFAR and the Global Fund. Comparing this counterfactual to the historical epidemic trajectory enables us to isolate the likely impact of U.S. investment. The two scenarios were developed as follows.

**4.1 The historical HIV response**

To date, the Global Fund and PEPFAR have funded treatment delivery, supported testing and counselling services, lower the cost of antiretroviral drugs, spearheaded voluntary medical male circumcision and behaviour change programs, and encouraged affected countries to increase their own domestic HIV spending. In our model, moving HIV-positive people onto ART lowers their rate of onward HIV transmission, and behavior change communication reduces the mean rate of changing partners. To model these historical interventions, parameters representing initiation years, rates of scale-up, and (in the case of ART) dependence on eligibility criteria were calibrated in our model against numbers on ART from national reports aggregated by UNAIDS [24] and self-reported data on sexual behaviors in Demographic and Health Surveys or equivalent sources [29–31]. Further details can be found in the recent paper by McGillen et al. [12].

**4.2 Counterfactual with no U.S. investment**

If the US had not contributed to and catalyzed the growing international HIV response in the late 1990s and early 2000s, the Global Fund might not have been created in 2002 and the Bush Administration would have failed to found PEPFAR in 2003. This is the counterfactual which we consider here in order to measure the impact of U.S. investments. We model domestic investments remaining flat at 2002 levels, the cost of ART remaining high, and a loss of any behavior change communications that began after the time of PEPFAR's founding.

The unit cost of treatment is held constant at a single value (both over time and across locations) in the 'real historical' scenario; this unit cost was derived by McGillen et al. [12] from the UNAIDS Fast Track modeling framework and set to $594 per person per year (which includes developmental synergies as per the Fast Track framework). For the counterfactual scenario, we set the (constant) unit cost of treatment at a higher value which approximates the level of cost that was typical in 2002 at the end of the pre-Global Fund/PEPFAR era. Estimates of the past cost of treatment leading up to 2002 vary widely depending upon context and the included externalities [32], with some estimates placing the then-standard triple therapy as high as $12,000 per patient per year excluding externalities [33]. The introduction of generics, catalyzed by the WHO Accelerating Access Initiative, enabled a large drop in cost prior to the founding of PEPFAR, and since then, an additional three-fold reduction in cost has been achieved with PEPFAR's influence. However, costs of service delivery, while difficult to quantify, are likely to have been much higher in 2002 than today, as service delivery in the early 2000s was based on secondary and tertiary care and the today it is more integrated into primary healthcare systems. We therefore estimate, for simplicity, that the total cost of treatment provision in 2002, including externalities, would have been approximately an order of magnitude greater than the current cost, or $5940 per patient per year.

We then continue to characterize the counterfactual by using these two (constant) unit cost estimates to calculate what relative population coverage of ART in a given country could have been attained in the absence of PEPFAR and the Global Fund, here denoted as
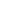
. Letting *A* denote the real historical level of population coverage of ART achieved in the country (facilitated at least in part by PEPFAR and the Global Fund), we have, for each modeled country,

where *c_p_* denotes the present cost of ART, *c*_2002_ the elevated cost of ART in 2002, *d*_2002_ the estimated domestic HIV expenditure in 2002, and *y*_p_ the most recent available total HIV expenditure from all sources combined. To estimate *d*_2002_, we extrapolate backward in time from later trends from UNAIDS [24] using logistic fits (Fig. S3).

As the U.S. has been the driving force behind historical behavior change programs such as condom distribution and promotion, we further assume that any behavior change initiatives that began after 2002 are likely to have been led by the Global Fund and PEPFAR, and hence these would have been less effective in the counterfactual scenario due to the absence of U.S. influence. We thus reduced by 50% any behavior change that began after 2002 in the modeled provinces. In provinces for which the best-fit parameter set included behavior change beginning prior to 2002, we did not reduce this in the counterfactual scenario.

**Figure S3: Estimated domestic HIV spending in 2002.** Backward extrapolation of domestic HIV spending data available from UNAIDS [24] to 2002 via logistic fitting.

5 Future prevention interventions and their unit costs

In the model framework, prevention interventions can be targeted to population groups to change their proximate determinants of risk and thereby impact the cumulative incidence across population groups. Prevention interventions considered here (currently available 'on the market') are oral PrEP, behavior change communications such as condom promotion, voluntary medical male circumcision, and universal testing and offer of treatment (UTT). UTT efforts will typically reach people out in communities who have HIV but are not yet ill enough to have sought care in a clinic setting. In the model, PrEP and voluntary medical male circumcision each reduce the per-partnership probability of HIV transmission, while behavior change communication reduces the mean rate of changing partners. UTT amplifies the number of HIV-positive people on ART, reducing onward transmission.

The maximum achievable population coverage is assumed to be 100% for all interventions. We have made assumptions around the intervention effectiveness and assigned unit costs in accordance with the UNAIDS Fast Track framework [1] (Table S2); costs were multiplied by a factor of 1.3 to account for developmental synergies, also in accordance with Fast Track. These unit costs are not intended to supercede the more detailed UNAIDS cost estimates, but are simplified costs derived from the Fast Track framework to answer new questions. Due to the present difficulty of comprehensive intervention costing on a detailed by-country basis, unit costs are assumed to be the same in all modeled countries. In the future, thanks to new data collection efforts, it will be possible to measure prevention properties in their local context, allowing cost estimates to be refined.

**Table S2: Available prevention interventions** and characteristics of their application to the modeled population groups. VMMC, voluntary medical male circumcision; BCC, behavior change communication; UTT, universal testing and offer of treatment; PrEP, pre-exposure prophylaxis; *, reduction in risk of HIV acquisition; †, reduction in risk of onward HIV transmission; ‡, per procedure; §, per person per year. Estimates are consistent with UNAIDS Fast Track [1] with unit costs derived in the paper by McGillen et al. [12]. UTT effectiveness is set at 85% to represent suboptimal long-term viral suppression that may result from loss to follow-up or development of drug resistance. Unit costs are in USD.

| Intervention | Effectiveness | Unit cost |
| --- | --- | --- |
| VMMC for HIV-negative uncircumcised men | 60% * | $88 ‡ |
| BCC for HIV-negative women in the general population | 20% * | $72 § |
| BCC for HIV-negative men in the general population | 20% * | $72 § |
| BCC for HIV-negative female sex workers | 50% * | $36 § |
| BCC for HIV-negative men who have sex with men | 50% * | $36 § |
| UTT for HIV-positive women in the general population | 85% † | $594 § |
| UTT for HIV-positive men in the general population | 85% † | $594 § |
| UTT for HIV-positive female sex workers | 85% † | $594 § |
| UTT for HIV-positive men who have sex with men | 85% † | $594 § |
| PrEP for HIV-negative women in the general population | 75% * | $123 § |
| PrEP for HIV-negative men in the general population | 75% * | $123 § |
| PrEP for HIV-negative female sex workers | 75% * | $123 § |
| PrEP for HIV-negative men who have sex with men | 75% * | $123 § |

6 Analysis of future U.S. policy options

The second phase of our analysis examines the consequences of potential changes to U.S. funding policy for the modeled epidemic future in sub-Saharan Africa. Our timeframe for this analysis is the fifteen-year period from 2017 to 2032. We explore a series of four scenarios: no further program expansion, increased U.S. funding, increased U.S. and domestic funding, and increased U.S. and domestic funding with optimal allocation. Financial and methodological details of these scenarios are discussed below.

**6.1 No further expansion of programs**

We consider a minimal-response case in which the present number of people on anti-retroviral therapy is maintained, but no further expansion of treatment or prevention programs is funded over the next 15 years. In the model, we control the flow of HIV-positive adults onto treatment such that the number of adults on ART established by 2016 is held fixed over all subsequent years. Historical behavior change intiatives are allowed to persist, but no introduction of new prevention programs is considered. The amount of funding this scenario would require is determined by multiplying the total number of adults on ART in the 15-year period by the unit cost of ART ($594 per person per year as in Section 5).

**6.2 Increased U.S. funding (with status quo otherwise)**

This scenario supposes that treatment and prevention initiatives will be scaled up in the future, with the U.S. increasing its investments in PEPFAR and the Global Fund by 10%. We assume a 'status quo' spending pattern whereby other international resources remain flat and funding is distributed across countries following present patterns, governments of affected countries grow their own HIV spending only in line with economic trends, and new prevention initiatives are rolled out nationally. Each of these conditions is implemented as follows.

*6.2.1 Projections for international spending*

For each country the current total U.S. funding contribution is the sum of the PEPFAR disbursement and U.S. portion of the Global Fund disbursement (Table S1). We increase these values by 10% and hold them constant over the 15 years of interest. For other international sources (including the non-U.S. portion of the Global Fund), we assume that funding will remain flat annually at the most recently reported values. This is plausible in light of the fact that, as the HIV/AIDS response begins to move out of the 'emergency' phase of the past decade and into long-term maintenance, commitment may be limited by competing global issues or new emerging health crises. We further assume that donors will not deviate from how they currently distribute funding across countries.

*6.2.2 Projected domestic HIV spending in line with economic trends*

Following a recent analysis by Resch et al. [18], we consider a 'status quo' scenario for domestic funding whereby the growth of HIV expenditure by governments of implementing countries is driven solely by macroeconomic trends. Current domestic public HIV expenditures (Table S1) form the core of our projections for future funding. For a given country, we can break down this expenditure, or the total government HIV/AIDS expenditure (GAE), into components of the gross domestic product (GDP) according to the formula

(1)

where *f*_GGE_ denotes the fraction of GDP going to general government expenditure; *f*_GHE_ the fraction of GGE going to health expenditure; and *f*_GAE_ the fraction of GHE going to AIDS-specific expenditure, calculated from the values of the other data items in the most recent available year. Subsequently we will denote the most recent available year by
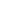
.

Data on GDP and government spending were obtained from the International Monetary Fund (IMF) [34]. Projections by the IMF extend from 2015 through 2021. We further project these estimates through 2031 by assuming that the rate of economic growth established by 2021 will remain constant annually thereafter. From the WHO Global Health Expenditure Database [35] we obtained historical estimates of government health expenditure (GHE) as a percent share of the GGE. Fig. S4 summarizes these domestic expenditure data in year
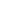
 by country.

Projecting into the future, we assume for this 'economic trend' scenario that the share of GDP spent (*f*_GGE_), the share of that spending going to health (*f*_GHE_), and the share of health spending going to HIV/AIDS (*f*_GAE_) will all be maintained at the status quo, such that the government AIDS expenditure (GAE) will increase only according to expected growth of GDP as projected by the IMF. Equation (1) thus becomes

**Figure S4: Domestic spending breakdown** in the most recent available data year. Gross domestic product (GDP, light blue), general government expenditure (GGE, mid-light blue), government health expenditure (GHE, mid-dark blue), and government AIDS expenditure (GAE, dark blue). Bars are nested to show that, for example, a government's spending on AIDS (GAE) comprises a portion of its total health expenditure (GHE). Note that the y-axis is on a log scale.

*6.2.3 National allocation of prevention interventions*

Before scaling up any future HIV prevention, we require that the treatment needs of each country are met to the greatest possible extent given the projected available funding. We determine how many people, in the absence of any prevention, need to be on ART in a given country in order for that country to successfully continue its established pattern of sevice scale-up, and how much this will cost. We then determine whether these needs can be met by the total HIV/AIDS funding available to that country or whether a funding deficit will remain. This ensures that the top funding priority captured in our model is always the offer of ART to all who present for care. Prevention interventions are then applied over and above this baseline level of treatment. Unit costs other properties of the interventions are as in Section 5 and Table S2. Existing coverage levels for behavior change communication and voluntary medical male circumcision depend on historical initiatives and the prevalence of traditional circumcision. UTT and PrEP are assumed to start from 2017.

Although the U.S. and Global Fund have recently advocated for prioritizing the geographies and populations in greatest need, it is not clear how successfully this has been translated into program strategies as yet. Kenya has changed its country program planning to reflect a county-centered (rather than national) strategy via County AIDS Strategic Plans, but this may be an exceptional case. We thus assume that for the majority of countries, a 'status quo' approach to prevention will comprise national-level programs that do not specifically target key at-risk population groups. For each country, we identify the prevention combination that returns the greatest impact (in terms of HIV infections averted) within the budget that is available for prevention in that country after treatment needs have been accounted for. Portfolio choices are constrained such that each intervention must be applied to all eligible population groups if it is used; for example, if PrEP is included in a portfolio, it must be offered to HIV-negative men and women in the general population, female sex workers, and men who have sex with men. Further details of this algorithm can be found in the recent paper by McGillen et al. [12], where it is referred to as the 'uniform' strategy.

**6.3 Increased U.S. and domestic funding**

In another scenario, governments of affected countries might increase their domestic HIV spending beyond the levels that would be dictated by economic trends. Other conditions—for U.S. spending, other international funds, and allocation of prevention interventions—remain as described in Section 6.2.

Resch et al. [18] present a series of targets toward which governments of implementing countries might increase their domestic HIV spending. Other spending targets have been offered, for example by Galarraga et al. [36] and Remme et al. [37], but many are tied to countries' historical performance, while those laid out by Resch et al. are intended to set normative yet optimistic standards for going forward. We consider an ambitious target which places countries' AIDS-specific spending into the context of the severity of their HIV/AIDS disease burden. It requires that the ratio of the HIV/AIDS share of health expenditure to the HIV share of the total disease burden meets or exceeds a value of 1/2. The disability life-adjusted year (DALY) is used as a marker of disease burden. We have obtained estimates of the share of total DALYs that were attributable to HIV/AIDS in 2013 (the most recent available year) in each country from the Institute for Health Metrics and Evaluation [38] (Table S3). Following Resch et al., the desired spending-to-DALY share ratio is set to 0.5 in order to be conservative and avoid an over-emphasis on AIDS, as it is well-established that distributing resources solely in proportion to national disease burden will not necessarily maximize health impact [39]. Moreover, the benchmark presents an achievable target; empirical data suggest that such a level of expenditure is feasible in low- and middle-income countries even with severe AIDS epidemics [18].

**Table S3: HIV/AIDS burden by country.** Percent of all disability-adjusted life years (DALYs) that were attributable to HIV/AIDS in 2013 according to the Institute for Health Metrics and Evaluation [38]. DRC=Democratic Republic of the Congo.

| Country | HIV DALY  share (%) | Country | HIV DALY  share (%) |
| --- | --- | --- | --- |
| Benin | 4.73 | Mozambique | 24.2 |
| Botswana | 32.3 | Nigeria | 7.34 |
| Burkina Faso | 2.69 | Rwanda | 8.66 |
| Cameroon | 11.5 | Sierra Leone | 4.95 |
| Congo | 12.2 | South Africa | 41.8 |
| DRC | 3.64 | Swaziland | 29.5 |
| Ethiopia | 6.18 | Tanzania | 13.2 |
| Kenya | 15.6 | Zambia | 20.0 |
| Mali | 1.97 | Zimbabwe | 26.7 |

To increase domestic spending to meet this benchmark, we modify Equation (1) such that we have:

Botswana, Burkina Faso, Ethiopia, Kenya, Mali, Rwanda, and Tanzania already meet or exceed this DALY share benchmark. Spending is close to the target in Benin, Cameroon, Congo, Nigeria, Swaziland, and Zimbabwe. The Democratic Republic of the Congo, Mozambique, Sierra Leone, South Africa, and Zambia would need to more than double their yearly AIDS spending in order to meet the target.

**6.4 Increased U.S. and domestic funding with optimal allocation**

The scenario in Section 6.3 can be made still more optimistic by supposing that the U.S. will lead a concerted effort to make the use of total prevention resources more responsive to epidemic dynamics. We assume that the increases in U.S. and domestic funding described previously will still occur (with other international funding remaining flat) such that the only difference between the previous scenario and this one is the way in which the funding is allocated in the model.

Rather than constraining the roll-out of prevention portfolios as previously, we relax the allocation algorithm to allow for specific provincial-level and population targeting. In each province, we enumerate all combinations of interventions—allowing each intervention to be applied independently to each different population group according to Table S2—and select combinations that provide the greatest health return in terms of infections averted for the lowest cost (the objective function). We then calculate the total financial envelope by summing the available resources over all countries, minus the total treatment needs. Defining a small increment of this total financial envelope, we award the increment to the province whose locally-optimal prevention portfolio produces the greatest health gains for that amount of money. The process is iterated until the entire financial envelope has been distributed. This allows prevention portfolios to compete within each province and the best choices of these to compete between all provinces, producing an allocation pattern whereby prevention is freely optimized to the local epidemiology. This approach is discussed further in the recent paper by McGillen et al. [12] where it is referred to as the 'focused' strategy.

7 Key assumptions and limitations of this study

Our financial estimates and projections are subject to the extent of publicly available data, and hence are incomplete or approximate for some countries and years. Our set of modeled countries overlaps with, but is not the same as, the set of PEPFAR focus countries, which might bias our conclusions. Moreover, while we have considered only sub-Saharan Africa here, PEPFAR and the Global Fund each support countries elsewhere in the world.

We have assumed that future funding will be used primarily for direct program costs of existing treatment and prevention methods, rather than being put into, for example, research and development of novel methods or health system strengthening. As discussed in Section 5, unit costs of prevention methods are assumed to be the same everywhere due to data limitations. Full fungibility of funds is assumed by our allocation algorithm, both over time and (in the case of optimal prevention) across country boundaries, with choices of intervention bundles then persisting over the 15-year intervention period.

The worst-case future scenario we have considered is a maintenance of current numbers on treatment without further expansion of programs. However, if severe funding cuts by major donors such as the U.S. were to occur, many HIV care centers could be forced to close, making it difficult to maintain provision of treatment even to existing patients.

Reliable data on female sex workers and men who have sex with men are scarce, and quantitative conclusions related to these key populations should be made with caution. Our integration of multiple data sources in the model calibration introduces additional uncertainty, and thus the model presents only one possible epidemic future that is consistent with historical trends. A comprehensive discussion of modeling limitations can be found in the paper by McGillen et al. [12].

8 References (continued from main text)

1. UNAIDS. **AIDS Info dashboard**. Geneva, Switzerland, 2017. URL: aidsinfo.unaids.org. [Accessed 31 Jan 2017].
2. Office of the U.S. Global AIDS Coordinator. **Obligation and Outlay Reports, 2009–2017** (Archived). Washington DC, USA, 2017. URL: 2009-2017.pepfar.gov/about/c24880.htm [Accessed 7 Apr 2017].
3. amfAR. **PEPFAR Country/Regional Operational Plans (COPs/ROPs) database**. Washington DC, USA, 2017. URL: copsdata.amfar.org [Accessed 7 Apr 2017].
4. Aidspan. **Aidspan Portal Workbench**. Nairobi, Kenya, 2017. URL: data.aidspan.org. [Accessed 7 Apr 2017].
5. Global Fund. **Core Pledges and Contributions List**. Geneva, Switzerland, 2017. URL: https://www.theglobalfund.org/documents/core/financial/Core_PledgesCon tributions_List_en/ (downloadable Excel file) [Accessed 7 Apr 2017].
6. USAID. **Demographic and Health Surveys (DHS)**. Washington DC, USA, 2015. URL: dhsprogram.com [Accessed 2 April 2017].
7. Human Sciences Research Council. **South African national HIV prevalence, incidence, and behaviour survey**. Pretoria, South Africa, 2012. URL: www.hsrc.ac.za/uploads/pageContent/4565/SABSSM%20IV%20LEO%20final.pdf [Accessed 31 Jan 2017].
8. Central Statistics Office. **2008 Botswana AIDS impact survey III: statistical report**. Ministry of Health: Gaborone, Botswana, 2009.
9. Rosen S and Long L. **How much does it cost to provide antiretroviral therapy for HIV/AIDS in Africa?** Health and Development Discussion Paper No. 9, Center for International Health and Development, Boston University, 2006. URL: bu.edu/av/iaen/research-library-1/docs/13387/Rosen%20Cost%20of%20ART %20in%20Africa.pdf [Accessed 6 March 2017].
10. UNAIDS. **Fact sheet: access to HIV treatment and care**. Geneva, Switzerland, 2004. URL: http://data.unaids.org/publications/fact-sheets04/fs_treatment_en.pdf [Accessed 6 March 2017].
11. International Monetary Fund. **World Economic Outlook database**. Washington DC, USA, 2016. URL: imf.org/external/pubs/ft/weo/2016/02/weodata/index.aspx [Accessed 31 Jan 2017].
12. WHO. **Global Health Expenditure Database**. Geneva, Switzerland, 2016. URL: who.int/health-accounts/ghed/en. [Accessed 31 Jan 2017].
13. Galarraga O, Wirtz V, Santa-Ana Tellez Y, Korenromp E. **Financing HIV programming: how much should low- and middle-income countries and their donors pay?** *PLOS One* 2013; **8**:e67565.
14. Remme M, Siapka M, Sterck O, Ncube M, Watts C, Vassall A. **Financing the HIV response in sub-Saharan Africa from domestic sources: moving beyind a normative approach**. *Soc Sci Med* 2016; **169**: 66–76.
15. Institute for Health Metrics and Evaluation. **Global Health Database**. Seattle, Washington, USA, 2017. URL: ghdx.healthdata.org [Accessed 13 Mar 2017].
16. William A. **Calculating the global burden of disease: time for a strategic reappraisal?** *Health Econ* 1999; **8**(1): 1–8.
